# Evaluation of Minerals Content of Drinking Water in Malaysia

**DOI:** 10.1100/2012/403574

**Published:** 2012-04-30

**Authors:** Azrina Azlan, Hock Eng Khoo, Mohd Aizat Idris, Amin Ismail, Muhammad Rizal Razman

**Affiliations:** ^1^Department of Nutrition and Dietetics, Faculty of Medicine and Health Sciences, Putra University of Malaysia (UPM), 43400 Serdang, Selangor, Malaysia; ^2^Laboratory of Halal Science Research, Halal Products Research Institute, Putra University of Malaysia (UPM), 43400 Serdang, Selangor, Malaysia; ^3^Institute for Environment and Development (LESTARI), National University of Malaysia (UKM), 43600 Bangi, Selangor, Malaysia

## Abstract

The drinking and mineral water samples obtained from different geographical locations had concentrations of the selected minerals lower than the standard limits, except for manganese, arsenic, and fluoride. The concentrations of manganese and arsenic in two mineral water samples were slightly higher than the standard international recommended limits. One mineral water sample had a fluoride concentration higher than the standard limits, whereas manganese was not detected in nine drinking and mineral water samples. Most of the selected minerals found in the tap water samples were below the international standard limits, except for iron and manganese. The concentrations of iron and manganese in the tap water samples were higher than the standard limits, which were obtained from one and three of the studied locations, respectively. The potable water obtained from various manufacturers and locations in Peninsular Malaysia is safe for consumption, as the minerals concentrations were below the standard limits prescribed by the Malaysian Food Regulations of 1985. The data obtained may also provide important information related to daily intake of these minerals from drinking water.

## 1. Introduction

Water, a renewable resource, is abundantly available in Malaysia. A total of 3000 mm of average annual rainfall produces the fresh water supply of this country. An estimated amount of 566 billion cubic metres (bcm) of rainwater runs off as surface flow and in the river systems each year [1]. The total demand for water usage is estimated to become 14 bcm by the year 2020, which equates to 12% of the total water available [2]. Approximately 99% of the water supply in Malaysia comes from rivers and streams in the country [3]. However, groundwater currently contributes 1% of the water required.

Generally, people living in developed countries have proper water supply at home. The quality of water received is clean and safe for consumption and can be consumed directly from the tap without posing any health threat [4]. The quality of drinking water in the United States, Europe, and Canada is acceptable according to the criteria set either by their governments or the World Health Organization (WHO) [5]. However, the United Nations has reported that 1.2 billion people are not able to access safe drinking water [1].

Nowadays, even with supplies of clean water to every home in big cities, most people in developing countries prefer to consume bottled drinking water, either locally bottled or imported. This preference for bottled water is due to the condition of tap water supplied to homes with an unacceptable taste and an unpleasant appearance in certain districts, which could be due to the taste of chlorinated tap water [6] or the contamination of tap water from leaking pipes and other forms of corrosion [7, 8]. In Malaysia, the volume of municipal water used by consumers for drinking is low [9]. However, most urban people preferred bottled water as an alternative to tap water [10]. Today, bottled drinking water has undergone a purification process, packed, and sold to the community [11].

Quality control of the water supply is monitored by a few agencies in the country. The Department of Environment is the agency that monitors the river basins in Malaysia to determine water quality in relation to major pollution sources [3], whereas state water authorities are responsible for the monitoring of raw water quality in the reservoirs at the intake point of the treatment plants [1]. Quality and safety of bottled drinking water (DW) and mineral water (MW) are monitored by the Ministry of Health, Malaysia, whereas state water authorities monitor the quality of tap water (TW) that is the source for the bottled DW [1]. However, geographical locations may affect the quality of portable water, which its mineral contents are very dependent on the mineral compositions of the soil and pollutants such as heavy metal.

In order to minimize mineral toxicity and maintain the wholesomeness of water consumption, the TW, MW, and DW that are intended for human consumption should comply with the mandated standard limits. The places of the water obtained may influence the mineral compositions of the water. No previous study has been performed to evaluate the mineral contents in both TW and bottled DW from different locations in Malaysia, especially the micromineral and heavy metal contents. Therefore, in this study, we aimed to determine the selected minerals in DW, MW, and TW obtained from various locations in Malaysia and compared with other studies from various countries. The selected minerals in different brands of bottled DW and MW obtained and TW collected from various locations were determined to investigate the quality of Malaysian DW.

## 2. Materials and Methods

### 2.1. Sample Preparation

A total of 24 bottled DW and MW samples from 22 brands were randomly collected from the shelf of selected local supermarkets and hypermarkets in Klang Valley, Malaysia. Stratified sampling was applied for the sample selection, where three bottles of each sample from each brand were purchased. Samples of the same brand were mixed well and analyzed as one sample. The bottled water samples comprised of 12 samples of MW and 12 samples of DW. All drinking water samples were purchased in sealed 500 mL plastic bottles. All bottles were kept sealed and refrigerated at 4°C until the time of analysis.

Demineralized one liter plastic containers were used for collection of TW samples. The bottles were preserved in a 1 : 1 nitric acid solution for 2 days and rinsed with demineralized water. The TW samples were randomly collected from different locations in the selected 12 states of Peninsular Malaysia. Three replicates of TW samples from each location were randomly collected from two different places in each of the identified state in Malaysia. The TW samples were collected from shops, schools, petrol stations, and housing areas. The replicate samples of one liter each were obtained from the tap after the water was left running for at least 5 min before sample collection.

The collected TW samples were stored in the de-mineralized containers, sealed, and transported to laboratory at refrigerated temperature of 4–6°C. The TW samples were filtered through 0.45 *μ*m pore diameter membrane filters. The pH of the filtrate was adjusted to pH 2.0 using a Toledo 320 pH meter (Mettler-Toledo Inc., Columbus, OH) with nitric acid immediately after filtration. All water samples were analyzed within 14 days from the day of collection, and no preservatives were added to any of the collected samples.

### 2.2. Sample Analysis

The DW, MW, and TW samples (100 mL) were analyzed for the minerals using a novAA 400 flame atomic absorption spectrophotometry (AAS) system (AnalytikJena, Jena, Germany) and a GBC 908AA graphite furnace AAS system (GBC, Victoria, Australia), whereas manganese (Mn) in the samples was analyzed using a Hewlett-Packard inductively coupled plasma-optical emission spectrophotometry system equipped with an auto sampler, an electrothermal vaporization, a laser ablation, an ultrasonic nebulizer, and a hydrite generation system (Wilmington, DE) as described by Rosborg et al. [12].

The EPA 600/4-91-0101 method [13] was applied for determination of minerals in the samples. Nonmetal mineral contents in DW, MW, and TW samples were analyzed using test kits purchased from Merck (Darmstadt, Germany) and measured using a Spectroquant NOVA 60 photometer (Merck, Darmstadt, Germany). All solutions were prepared using double-distilled acids and type I water from laboratory reagent-grade water systems.

A flame AAS method was used to determine the concentrations of sodium (Na), magnesium (Mg), potassium (K), calcium (Ca), iron (Fe), copper (Cu), and zinc (Zn) in the DW, MW, and TW samples. For the flame AAS method, minerals of the samples were analyzed by applying several conditions as described in Table 1. A graphite furnace AAS was used to determine chromium (Cr), nickel (Ni), arsenic (As), cadmium (Cd), and lead (Pb). The specific atomic absorption was set (Table 1), and absorbance was obtained. Certified test kits purchased from Merck Chemicals (Darmstadt, Germany) were used to determine the nonmetal mineral contents in the water samples and measured with a Spectroquant NOVA 60 photometer.

High-purity mineral standards (CertiPUR grade) were purchased from Merck Chemicals (Darmstadt, Germany) and were used for calibration and quality control. Certified reference materials were obtained from the Commission of European Communities (CRM-616 and CRM-617) for the determination of the accuracy of the analytical method. Ultrapure nitric and hydrochloric acids were obtained from Seastar Chemicals (Sidney, BC, Canada). The quality of the data was evaluated by comparing the selected mineral concentrations of DW, MW, and TW samples with CRM values. For precision analysis, the relative standard deviation (RSD) (%) was obtained from the values generated by the AAS novAA 400 software. Data were statistically analyzed and are reported as mean, maximum, and minimum values.

## 3. Results and Discussion

Concentrations of the selected minerals in 14 DW, 13 MW, and 24 TW samples are presented in Tables 2–4, respectively. The selected minerals determined were Na, Mg, K, Ca, Fe, Cu, Zn, Cr, Mn, Ni, As, Cd, Pb, F, Cl, NO_3_, and SO_4_. The concentrations of the selected minerals found in the DW, MW, and TW samples were compared with national and international standard limits. The selected minerals in the studied water were also evaluated by comparing to other international studies.

Based on the results, the selected minerals in the DW, MW, and TW samples obtained from different geographical locations were varied. It shows that geographical location greatly influenced the mineral compositions of the ground water or portable water, where the most important factor is environmental pollution. Besides, the studied water samples also contained clinically important levels of macro- and microminerals.

### 3.1. Mineral Concentrations in DW Samples

Among the DW samples studied, DW 11, DW 13, and DW 14 had the highest concentrations of Na (10.68 mg/L) and Ca (1.67 mg/L), Mg (12.29 mg/L) and K (3.78 mg/L), and Cu (7.3 *μ*g/L) (Table 2), respectively. The concentrations of Fe (41.30 *μ*g/L), Cr (2.56 *μ*g/L), and Mn (31.0 *μ*g/L) were highest in DW 3, DW 1, and DW 9, respectively. For the heavy metal, the highest concentrations of As (1.68 *μ*g/L) and Pb (3.92 *μ*g/L) were found in DW 11, whereas Cd (0.85 *μ*g/L) was the highest in DW 1.

In some of the DW samples, trace concentrations of Mg, K, Ca, Zn, Cr, As, and Pb were detected using the AAS method. DW 1, DW 2, DW 3, and DW 12 samples had the lowest concentrations of Fe (17.2 *μ*g/L), Cu (0.2 *μ*g/L), Ni (0.13 *μ*g/L), and Cd (0.38 *μ*g/L), respectively. Mn was not detected in eight DW samples. DW 3, DW 7, DW 11, and DW 13 had the highest concentrations of F (0.71 mg/L), Cl (42.41 mg/L), NO_3_ (2.27 mg/L), and SO_4_ (52.02 mg/L). However, DW 4 and DW 7 had similar Cl concentrations. Concentrations of Cl found in seven DW samples were lower than 2.5 mg/L. Trace amounts of SO_4_ were also found in DW 1 and DW 3, whereas DW 9 and DW 1 had the lowest concentrations of F (0.05 mg/L) and NO_3_ (0.11 mg/L).

### 3.2. Mineral Concentrations in MW Samples

Among the MW samples studied, MW 12 had the highest concentrations of Mg (24.03 mg/L), Na (23.68 mg/L), and K (5.95 mg/L). MW 5, MW 6, MW 12, MW 4, and MW 10 had the highest concentrations of Ca (25.06 mg/L), Fe (60.5 *μ*g/L), Cu (16.9 *μ*g/L), Zn (24.3 *μ*g/L), and Mn (68.0 *μ*g/L) (Table 3), respectively. For the toxic elements, the highest concentrations of Ni (6.88 *μ*g/L), As (13.51 *μ*g/L), Cd (0.45 *μ*g/L), and Pb (1.25 *μ*g/L) were found in MW 2, MW 6, MW 5, and MW 9, respectively. On the other hand, MW 1, MW 5, MW 2, MW 3, MW 11, and MW 12 had the lowest concentrations of Na (2.40 mg/L), Mg (0.72 mg/L), K (0.60 mg/L), Ca (0.59 mg/L), Cu (1.5 *μ*g/L), and Zn (0.4 *μ*g/L), respectively. Trace concentrations of Cr were detected in all MW samples, whereas trace amounts of Fe and Pb were found in some of the MW samples. Mn was not detected in MW 1.

In this study, the concentrations of the selected mineral in MW samples were relatively higher than in DW samples, especially for F and SO_4_. MW 5 had the highest concentrations of Cl (58.58 mg/L) and NO_3_ (2.84 mg/L), whereas MW 6 and MW 7 had the highest concentrations of F (2.00 mg/L) and SO_4_ (18.74 mg/L), respectively. The lowest concentrations of F (trace), Cl (27.16 mg/L), and NO_3_ (0.12 mg/L) were detected in MW 5, MW 3, and MW 10, respectively. However, SO_4_ was not detected in MW 2.

### 3.3. Mineral Concentrations in TW Samples

Among the TW samples studied, TW 2 had the highest concentrations of Mg (3.14 mg/L), TW 20 had the highest concentration of Ca (14.48 mg/L), whereas TW 23 had the highest concentrations of K (6.61 mg/L) and Fe (0.33 mg/L) (Table 4). The highest concentrations of Na (15.03 mg/L), Cu (0.26 mg/L), Zn (0.36 mg/L), and Mn (0.1 mg/L) were found in TW 24, TW 20, TW 10, and TW 14, respectively. On the other hand, the lowest concentrations of Mg (0.07 mg/L) and Ca (0.54 mg/L) were found in TW 7, Na (1.22 mg/L) and Cu (0.01 mg/L) in TW 8, K (0.90 mg/L) in TW 21, Fe (0.02 mg/L) in TW 17, Zn (0.002 mg/L) in TW 6, and Mn (0.004 mg/L) in TW 3. Cr was not detectable in all TW samples studied, except TW 24.

Heavy metal (Ni, As, Cd, and Pb) concentrations of TW samples were determined. Results indicate that Cd (0.85 *μ*g/L) and Pb (3.80 *μ*g/L) were found to be highest in TW 23, whereas TW 20 had the highest concentrations of Ni (2.63 *μ*g/L). Pb was not detected in TW 1 to TW 22. The lowest heavy metal concentrations found in the TW samples ranged from not detectable to 0.36 *μ*g/L. High nonmetal minerals determined in the TW samples were F (0.71 mg/L), Cl (48.04 mg/L), NO_3_ (11.00 mg/L), and SO_4_ (31.22 mg/L) as found in TW 13, TW 23, TW 13, and TW 18, respectively. The lowest Cl (5.02 mg/L) and SO_4_ (1.06 mg/L) concentrations were found in TW 8, lowest F (0.14 mg/L) concentration in TW 20, and lowest NO_3_ (0.33 mg/L) concentration was found in TW 22.

### 3.4. Quality Control

CRM-616 and CRM-617 were used to determine the accuracy of the analysis. The optimum working ranges and standard working ranges for the studied minerals are shown in Table 1. RSD of the selected mineral concentrations in DW, MW, and TW samples are shown in Table 5. The detection and determination limits for the selected minerals in the water samples studied were 0.001 to 0.067 mg/L.

Mineral concentrations below the detection limit were considered not detectable [14]. The determination limit of Ca analyzed using flame AAS was the highest (0.067 mg/L), followed by Na (0.058 mg/L), Fe (0.046 mg/L), Mg (0.033 mg/L), and K and Cu (0.010 mg/L). The detection limit for Zn was the lowest compared to the other minerals. The test kits that were used to determine the nonmetal mineral contents have the ranges shown in Table 1.

RSDs of the mineral concentrations in DW and MW samples are shown in Table 5. RSD (%) for other minerals were not determined. Results show that the RSD of Na for all studied samples was less than 2.0%, except for DW1 (5.2%). The RSDs for Mg, K, Ca, and Zn in most of the DW and MW samples were also less than 2.0%, but the RSDs for the other minerals in the studied samples were within the range of 2–17%. The analyses of minerals in DW and MW samples with RSDs less than 8% are considered precise [15]. However, some of the analyzed samples have RSDs ranging from 8% to more than 17%. The high RSDs may be due to poor sensitivity of the instrument (AAS) used.

Results show that the RSDs of Na, Mg, K, and Ca concentrations in TW samples were less than 2.0%. Most of the RSDs for Fe, Cu, and Zn concentrations in TW samples were less than 8%, except for Ca, Fe, and Cu in a few TW samples that have RSDs ranging from 8–17%. The acceptable RSD range is between 2–8% [15], where most of the studied samples have RSDs within this acceptable range with some exceptional RSD values.

### 3.5. Comparative Assessment on Variation of Selected Minerals in DW, MW, and TW Samples according to the National and International Standard Limits

The studied minerals in all DW samples were below the standard limits recommended by the WHO Guidelines for Drinking Water Quality 2006 [16], the Council Directive 98/83/EC on the quality of water intended for human consumption [17], the United States Environmental Protection Agency (USEPA) Drinking Water Contaminants Regulations [18], and the Malaysian Food Regulations 1985-360B for packaged drinking water [19] (Table 2). No national and international standard limits were available for Ca and K in DW. Some of the USEPA recommendations are secondary standards which are nonenforceable. These include Fe, Cu, Mn, F, Cl, and SO_4_.

The selected concentrations of mineral in MW samples were relatively higher than those in DW samples. Mn concentrations in MW 6 and MW 10 were higher than the maximum permitted concentration recommended by the EU Standards [17] and the USEPA [18]. The concentrations of As in MW 6 and MW 7 were also higher than the maximum permitted concentrations recommended by the WHO [16], the EU Standards [17], and the USEPA [18], whereas the F concentration in MW 6 was higher than the maximum permitted concentration recommended by the WHO [16] and the EU Standards [17]. In this study, MW 6 had both As, and F concentrations higher than the standard limits. Similar observations have been reported in Mexico, where some of the drinking water in the Mexican market had high As and F levels, where the concentrations were above the levels recommended by the Mexican DW standard limits [20]. Although three MW samples had concentrations of Mn, As and F higher than international recommended values, consumption of these MW is still allowed, as it complied with the standard limits prescribed in the Malaysian Food Acts 1983 and Regulations 1985-360A for natural mineral water.

Higher level of Mn found in MW samples may be due to contamination from the water source. However, Mn concentrations in most DW and MW samples were well below the maximum permitted level of the WHO Guidelines 2006 and the Malaysian Food Regulations 1985. MW 6 had high concentrations of Mn and F, where the concentrations were lower than the values recommended by the WHO [16] and the USEPA [18] for Mn and F, respectively. The concentrations of these minerals, however, were still lower than the standard limits set by the Malaysian Food Regulations 1985-360A [19]. In TW samples, Fe concentrations were below the maximum permitted level recommended by EU Standards [17], WHO [16], and USEPA [18]. Mn concentrations in these samples were below the maximum permitted level recommended internationally. No specific reference maximum permitted levels were available for Mg, K, and Ca. For other minerals, the concentrations in TW samples were well below the maximum permitted level recommended by the international standard limits (Table 4).

Generally, bottled DW is safe for consumption, as the manufacturers have complied with the regulations enforced by the Malaysian government. In the present study, DW samples have the concentration of minerals lower than the standard limits approved nationally and internationally. This is because DW was obtained from TW that had been filtered or physically treated by the local water authorities and had subsequently been retreated in the factory by decantation or filtration. Some of the bottled DW in Malaysia are also subjected to a reverse osmosis process that guarantees it to have higher purity than water that is only filtered, whereas MW is obtained from groundwater. Groundwater normally has a higher content of dissolved solids than surface waters (i.e., lakes, rivers). This could be the reason for why MW contained higher concentrations of the mineral as compared to DW.

### 3.6. Mineral Compositions in Drinking Water from Previous Studies

Mineral compositions in drinking water have been studied worldwide since the last century. The concentrations of minerals in 33 different brands of bottled waters on the Swedish market were relatively higher than the concentrations found in the DW and MW samples studied in this investigation (data not shown), except for Cd, Pb, and NO_3 _[21]. In that study, however, large variations were found for Ca, Mg, Na, K, and Cl. Minerals in bottled drinking water (type of water not mentioned) from Egypt have been studied by Saleh et al. [22], where higher essential mineral concentrations and lower levels of heavy metal were detected as compared to the DW and MW samples studied here.

Mineral compositions in 66 natural mineral water sources from 19 Asian and European countries have also been analyzed [23]. Results from their study have shown that some of the mineral compositions did not comply with international guidelines for drinking water. Based on their study, mineral water collected from Malaysia had concentrations of F and NO_3_ higher than the concentrations found in this present study. However, higher Cl and SO_4_ were found in our MW samples compared to MW samples studied by Lau and Luk [23], whereas Na, Mg, K, and Ca concentrations in the present study were comparable to their study.

Mineral compositions in the DW and MW samples were compared with the Turkish, Canadian, Italian, and Japanese studies, as shown in Table 6. The mineral concentrations in DW samples were lower than the concentrations reported by Azoulay et al. [25], Chiba et al. [26], and Güler and Alpaslan [24], with some exceptions. The concentrations of trace minerals such as Fe, Cu, Zn, Cr, Mn, and other heavy metals in the studied DW samples were higher than values reported by Chiba et al. [26] and Güler and Alpaslan [24]. The mineral concentrations in MW samples were comparable with previous studies [24, 25], except Mn and Cl, where the mean concentrations were more than 10 times higher than the values reported by Güler and Alpaslan [24]. A study by Naddeo et al. [27] showed that bottled mineral water in Italy had higher mean concentrations of mineral than the studied MW samples, except for Cu and Ni, which were in trace amounts.

In TW samples, the concentrations of macrominerals determined were found to be lower than the concentrations found in tap water as reported by Chiba et al. [26] and Saleh et al. [22] (Table 6). However, the concentration of K in the studied TW samples was higher than the concentration found in the tap water obtained from Japan and Kazakhstan [26]. The concentrations of microminerals in TW samples were lower than the concentrations found in Egypt's tap water samples, except for Cu and Mn. As reported by Chiba et al. [26], Japanese's tap water samples had a low concentration of Fe, whereas Khazakhstans's tap water samples contained a high concentration of Zn. The studied TW samples had higher heavy metal concentrations compared to Egypt's tap water samples, except for the concentration of Ni. Moreover, the Cl and SO_4_ concentrations found in the studied TW samples were lower than those in Egypt's tap water.

## 4. Conclusions

This study evaluated the selected macrominerals, microminerals, heavy metals, and other inorganic elements in the water commonly consumed by Malaysian. All mineral concentrations in the DW, MW, and TW samples studied were found to be below the national and international standard limits, except for Mn in MW 6 and MW 10, As in MW 6 and MW 7 and Fe in TW 23. Mn concentrations in some of the studied samples were higher than the standard limits recommended by the EU Standards and USEPA Regulations. Cr and Pb were not detected in most of the TW samples studied, except Cr in one of the samples and Pb in two samples. Cr was also found in trace amounts in all MW samples, whereas trace amounts of other minerals were detected in some of the samples studied. F concentration in MW 6 was higher than the maximum permitted level recommended by the WHO Guidelines 2006 and the EU Standards 1998. As TW is an important source of mineral intake in the rural areas, high level of heavy metals in the water may pose adverse health effects to the populations. The findings of this study suggest that regular determination of minerals in bottled DW, MW, and TW is important to prevent the occurrence of mineral toxicity due to drinking these water.

## Figures and Tables

**Table 1:** AAS analytical conditions for determination of minerals in drinking water and mineral water samples.

Element	Wavelength (nm)	Slit width (nm)	Nebulizer rate (mL/min)	Optimum working range (*μ*g/mL)	Standard working range (*μ*g/mL)	Sensitivity (*μ*g/mL)	CRM values (mg/mL)
Na	589.0	0.5	5.0	0.18–0.7	10–1000	0.004	14.3–61.6
Mg	285.2	0.5	5.0	0.1–0.4	10–1000	0.003	7.32–23.8
K	766.5	0.5	5.0	0.4–1.5	10–1000	0.008	0.5–9.67
Ca	422.7	0.5	5.0	1–4	10–1000	0.02	14.3–39.9
Fe	248.3	0.2	5.0	2–9	1–50	0.05	0.05–0.21
Cu	324.7	0.5	5.0	1–5	1–50	0.025	—
Zn	213.9	0.5	5.0	0.4–1.5	1–50	0.008	—
Cr	357.9	0.2	5.0	2–15	1–50	0.05	—
Mn	279.5	0.2	5.0	1–3.6	1–50	0.02	0.02–0.05
Ni	232.0	0.2	5.0	1.8–8.0	1–5	0.04	—
As	193.7	1.0	5.0	30–190	1–5	0.64	—
Cd	228.8	0.5	5.0	0.2–1.8	1–5	0.009	—
Pb	283.3	0.5	5.0	7.0–460	1–5	0.16	—
Fl	—	—	—	—	0.1–20.0^a^	—	—
Cl	—	—	—	—	2.5–60.0^a^	—	26.4–50.2
NO_3_	—	—	—	—	0.1–25.0^a^	—	25.8–50.7
SO_4_	—	—	—	—	1.0–35.0^a^	—	27.0–57.9

^a^Concentration of minerals in mg/L.

**Table 2:** Mineral concentrations in drinking water (DW) samples and the standard limits recommended by Malaysian Food Regulations and international regulations.

Drinking water	Na	Mg	K	Ca	Fe^a^	Cu^a^	Zn^a^	Cr^a^	Mn^a^	Ni^a^	As^a^	Cd^a^	Pb^a^	F	Cl	NO_3_	SO_4_
DW 1	0.01	Tr	Tr	Tr	17.2	1.7	Tr	2.56	ND	1.04	0.47	0.85	3.80	0.24	36.33	0.11	Tr
DW 2	0.31	Tr	0.11	Tr	23.4	0.2	0.6	0.32	ND	0.22	0.15	0.50	1.50	0.21	39.28	0.28	2.23
DW 3	0.64	Tr	0.13	Tr	41.3	1.0	0.8	Tr	ND	0.l3	Tr	0.78	1.80	0.71	35.12	0.33	Tr
DW 4	Tr	Tr	Tr	Tr	42.1	0.6	2.7	Tr	ND	0.49	0.17	0.48	1.57	Tr	42.36	0.31	4.15
DW 5	0.31	Tr	0.14	Tr	35.5	0.4	1.8	Tr	1.0	0.27	0.17	0.49	2.34	0.30	42.24	0.45	1.05
DW 6	0.38	0.003	0.38	0.15	37.9	1.2	1.4	Tr	ND	0.67	0.14	0.40	0.66	0.10	38.17	0.13	3.04
DW 7	0.30	Tr	0.86	0.02	28.7	1.0	1.8	Tr	ND	0.45	0.35	0.41	0.51	0.19	42.41	0.26	3.00
DW 8	Tr	Tr	Tr	Tr	43.3	2.8	2.0	Tr	2.0	2.09	0.25	0.43	Tr	0.19	Tr	0.12	2.03
DW 9	0.84	Tr	0.001	Tr	37.0	3.6	1.4	Tr	31.0	0.36	0.02	0.38	Tr	Tr	Tr	0.23	1.04
DW 10	0.67	0.03	0.60	0.18	36.7	4.1	1.5	Tr	15.0	0.26	Tr	0.43	0.28	0.27	Tr	0.32	6.05
DW 11	10.68	9.03	3.43	1.67	38.4	5.3	1.3	1.51	11.0	0.38	1.68	0.49	3.92	0.32	Tr	2.27	3.00
DW 12	1.48	0.45	1.38	0.30	30.2	5.7	0.3	Tr	ND	0.31	0.36	0.38	Tr	0.43	Tr	0.12	13.0
DW 13	9.48	12.29	3.8	1.64	36.6	6.9	0.5	0.19	12.0	0.36	1.11	0.42	1.34	0.18	Tr	1.36	52.02
DW 14	6.01	3.71	3.29	1.10	34.8	7.3	2.3	0.96	ND	0.64	0.39	0.42	0.24	0.19	Tr	1.52	1.01
*WHO 2006*	200	—	—	—	300	2000	—	50	500	20	10	3	10	1.5	250	50	500
*EU 1998*	200	—	—	—	200	2000	—	50	50	20	10	5	10	1.5	250	50	250
*USEPA 2009*	—	—	—	—	300	1300	5000	100	50	—	10	5	15	4	250	10	250
*MR 1985*-360B	200	150	—	—	300	1000	5000	50	100	—	50	5	50	1.5	250	10	400

All data were presented as mean of three replicates (mg/L). ^a^Concentration of minerals in *μ*g/L. Tr: trace; ND: not detected; WHO: World Health Organization Guidelines; EU: European Union Standards; USEPA: United States Environmental Protection Agency Drinking Water Contaminants Regulations; MR: Malaysian Food Regulations.

**Table 3:** Mineral concentrations in mineral water (MW) samples and the standard limits recommended by Malaysian Food Regulations and international regulations.

Mineral water	Na	Mg	K	Ca	Fe^a^	Cu^a^	Zn^a^	Cr^a^	Mn^a^	Ni^a^	As^a^	Cd^a^	Pb^a^	F	Cl	NO_3_	SO_4_
MW 1	2.40	2.84	1.15	3.17	16.7	12.3	4.1	Tr	ND	2.36	Tr	0.41	Tr	0.13	28.34	2.21	8.05
MW 2	3.58	2.70	0.60	1.65	17.4	10.1	3.3	Tr	19.0	6.88	0.03	0.37	0.09	0.14	28.02	0.34	ND
MW 3	3.55	6.67	4.46	0.59	19.2	11.6	1.4	Tr	35.0	0.74	0.39	0.44	0.33	0.30	27.16	0.72	5.35
MW 4	3.21	2.28	2.96	4.56	17.3	12.6	24.3	Tr	39.0	0.91	0.09	0.45	Tr	0.22	29.47	0.76	12.34
MW 5	21.18	0.72	2.53	25.06	15.9	12.2	12.7	Tr	30.9	3.49	0.27	0.45	0.69	Tr	58.58	2.84	13.97
MW 6	19.86	1.10	3.02	3.34	60.5	13.0	3.4	Tr	67.0	0.42	13.51	0.41	0.44	2.00	30.03	0.77	12.03
MW 7	9.80	3.00	3.61	3.32	3.0	14.8	2.2	Tr	4.0	0.74	12.71	0.36	Tr	0.27	31.23	0.52	18.74
MW 8	8.86	3.42	1.37	1.65	0.1	14.3	1.2	Tr	6.0	0.28	0.28	0.36	Tr	0.15	34.17	0.54	14.45
MW 9	7.70	2.31	2.52	4.66	Tr	15.1	0.8	Tr	39.0	0.58	2.65	0.39	1.25	0.78	35.53	0.28	10.12
MW 10	7.80	2.28	2.49	4.43	Tr	16.4	1.2	Tr	68.0	0.49	2.87	0.37	0.28	0.70	34.04	0.12	3.23
MW 11	10.37	12.00	3.64	1.71	Tr	1.5	0.7	Tr	30.0	0.71	0.72	0.37	0.34	0.18	37.98	2.14	4.05
MW 12	23.68	24.03	5.95	2.40	Tr	16.9	0.4	Tr	46.0	0.83	7.70	0.37	Tr	0.29	37.34	1.92	15.04
MW 13	10.24	12.61	3.53	1.49	Tr	15.2	6.6	Tr	26.0	1.13	0.40	0.43	Tr	0.17	36.03	1.95	4.20
*WHO 2006*	200	—	—	—	300	2000	—	50	500	20	10	3	10	1.5	250	50	500
*EU 1998*	200	—	—	—	200	2000	—	50	50	20	10	5	10	1.5	250	50	250
*USEPA 2009 *	—	—	—	—	300	1300	5000	100	50	—	10	5	15	4	250	10	250
*MR 1985*-360A	—	—	—	—	—	1000	5000	50	2000	—	50	10	50	2	—	45	—

All data were presented as mean of three replicates (mg/L). ^a^Concentration of minerals in *μ*g/L. Tr: trace; ND: not detected; WHO: World Health Organization Guidelines; EU: European Union Standards; USEPA: United States Environmental Protection Agency Drinking Water Contaminants Regulations.

**Table 4:** Mineral concentrations in tap water (TW) samples and the standard limits recommended by international regulations.

Tap water	Na	Mg	K	Ca	Fe^a^	Cu^a^	Zn^a^	Cr^a^	Mn^a^	Ni^a^	As^a^	Cd^a^	Pb^a^	F	Cl	NO_3_	SO_4_
TW 1	11.23	3.14	2.48	4.2	0.06	0.06	0.02	ND	0.01	1.39	0.49	0.38	ND	0.31	21.01	0.61	14
TW 2	4.88	4.11	3.0	23.1	0.03	0.09	0.03	ND	0.02	1.26	6.14	0.37	ND	0.24	6.23	0.64	17.12
TW 3	2.99	1.31	2.49	6.04	0.05	0.04	0.02	ND	0.004	0.68	0.33	0.39	ND	0.39	9	0.62	19.23
TW 4	2.82	0.95	2.31	2.01	0.06	0.02	0.004	ND	0.03	1.91	0.38	0.37	ND	0.3	6.08	0.82	14.16
TW 5	2.15	0.56	2.43	9.01	0.03	0.03	0.005	ND	0.03	0.36	0.16	0.36	ND	0.49	8	0.83	6.14
TW 6	1.95	0.45	2.45	5.36	0.03	0.02	0.002	ND	0.02	0.8	0.26	0.37	ND	0.29	10.11	1.61	6.11
TW 7	1.8	0.07	1.35	0.54	0.06	0.02	0.01	ND	ND	0.97	0.24	0.39	ND	0.38	5.02	0.65	5.04
TW 8	1.22	0.45	1.22	2.61	0.09	0.01	0.01	ND	0.01	0.58	0.42	0.36	ND	0.58	5.02	0.83	1.06
TW 9	3.74	0.43	3.38	3.6	0.03	0.06	0.01	ND	0.004	0.52	0.48	0.37	ND	0.2	8.04	1.31	20
TW 10	2.28	0.39	2.15	4.28	0.09	0.07	0.36	ND	0.04	0.85	0.22	0.38	ND	0.66	14	0.94	5.4
TW 11	3.04	0.4	2.36	1.84	0.09	0.08	0.06	ND	0.01	0.72	0.46	0.4	ND	0.58	11.32	0.92	9.09
TW 12	4.95	0.48	3.75	3.68	0.03	0.13	0.02	ND	0.01	0.53	0.95	0.39	ND	0.47	16.12	1.93	4.11
TW 13	3.84	0.25	3.04	1.99	0.03	0.06	0.02	ND	0.03	0.8	1.24	0.38	ND	0.71	16	11	7.02
TW 14	3.08	0.64	4.59	10.49	0.03	0.04	0.004	ND	0.1	0.52	0.31	0.46	ND	0.69	27.05	0.91	6.06
TW 15	5.19	1.12	4.87	3.27	0.03	0.05	0.005	ND	0.03	0.64	0.34	0.44	ND	0.34	37.07	1.52	5.01
TW 16	4.15	1.24	4.81	6.45	0.07	0.2	0.08	ND	0.06	0.85	1.1	0.43	ND	0.19	26.02	0.63	7.12
TW 17	3.57	1.35	6.02	7.61	0.02	0.08	0.02	ND	0.03	1.65	0.36	0.38	ND	0.34	29	1.14	17.34
TW 18	3.48	2.13	5.82	12.1	0.06	0.1	0.1	ND	0.05	1.53	0.51	0.4	ND	0.68	28.04	1.12	31.22
TW 19	1.61	0.3	2.15	9.44	0.03	0.11	0.01	ND	0.05	0.26	0.32	0.37	ND	0.47	29.05	0.74	10.42
TW 20	5.21	2.03	2.96	14.48	0.05	0.26	0.02	ND	ND	2.63	0.53	0.38	ND	0.14	34.19	0.71	8.6
TW 21	2.05	0.28	0.9	1.11	0.06	0.13	0.004	ND	ND	0.75	0.2	0.38	ND	0.15	25.01	0.53	ND
TW 22	2.13	0.29	0.97	3.2	0.03	0.12	0.004	ND	0.03	0.43	0.26	0.39	ND	0.2	27	0.33	6.04
TW 23	10.84	2.52	6.61	11.2	0.33	0.13	0.03	ND	ND	0.71	0.87	0.85	3.8	0.2	48.04	0.34	9.07
TW 24	15.03	2.53	4.62	12.02	0.04	0.14	0.05	0.001	0.05	0.42	2.76	0.5	1.5	0.23	38.23	2.72	15.33
*WHO 2006*	200	—	—	—	300	2000	—	50	500	20	10	3	10	1.5	250	50	500
*EU 1998*	200	—	—	—	200	2000	—	50	50	20	10	5	10	1.5	250	50	250
*USEPA 2009 *	—	—	—	—	300	1300	5000	100	50	—	10	5	15	4	250	10	250

All data were presented as mean of three replicates (mg/L). ^a^Concentration of minerals in *μ*g/L. Tr: trace; ND: not detected; WHO: World Health Organization Guidelines; EU: European Union Standards; USEPA: United States Environmental Protection Agency Drinking Water Contaminants Regulations.

**Table 5:** Percentage of relative standard deviation (RSD) of minerals in drinking water (DW), mineral water (MW), and tap water (TW) samples.

Sample	Na	Mg	K	Ca	Fe	Cu	Zn	Cr	Ni	As
DW 1	5.2	6.3	—	28.7	31.7	7.1	1.8	52.0	0.3	34.8
DW 2	1.1	—	2.2	—	35.2	7.5	3.8	13.3	37.2	17.1
DW 3	0.4	—	1.4	—	19.3	3.9	2.6	27.6	20.0	44.7
DW 4	—	—	—	—	28.4	9.7	0.3	4.6	27.1	22.8
DW 5	0.6	—	2.0	—	8.7	5.5	2.0	8.1	39.5	38.0
DW 6	0.9	3.6	1.5	0.3	12.8	10.1	4.4	1.1	81.6	40.9
DW 7	0.7	12.0	1.0	7.1	3.7	4.4	1.3	30.2	18.6	3.8
DW 8	—	—	—	—	11.2	4.3	3.9	12.2	15.2	4.2
DW 9	1.1	—	44.1	—	5.2	9.8	1.4	8.1	15.7	19.7
DW 10	0.4	2.3	1.1	3.2	6.6	4.1	1.8	12.2	65.5	30.6
DW 11	0.6	3.2	0.1	0.7	9.3	4.3	0.6	—	35.5	—
DW 12	0.3	1.2	0.2	2.2	3.7	3.9	1.6	33.1	25.2	—
DW 13	0.6	1.8	0.4	0.3	5.1	4.5	0.6	2.1	10.8	22.2
DW 14	0.6	0.3	1.0	0.7	9.6	7.7	2.6	6.4	31.9	3.7
MW 1	1.2	1.2	0.7	1.1	9.1	2.5	2.2	18.1	12.5	19.5
MW 2	1.3	0.7	1.1	1.2	15.7	2.2	1.4	25.6	5.9	3.5
MW 3	0.9	5.9	1.1	7.0	6.8	3.3	1.1	14.5	37.6	28.3
MW 4	0.6	0.6	0.4	1.1	16.6	2.6	0.5	2.6	24.2	13.2
MW 5	0.7	0.9	2.2	0.2	3.8	6.9	0.8	5.9	2.6	12.4
MW 6	0.4	0.5	1.5	1.7	6.7	4.3	0.8	13.5	39.2	3.0
MW 7	0.8	0.7	1.1	2.1	12.0	3.3	1.0	4.1	15.8	0.6
MW 8	0.6	0.5	0.3	3.9	2.5	1.7	1.5	2.1	10.6	24.5
MW 9	0.4	0.6	0.5	2.1	16.6	4.0	0.9	4.6	10.9	15.2
MW 10	0.2	0.3	1.1	3.2	11.1	3.7	0.7	7.5	44.6	0.8
MW 11	0.3	1.9	2.9	1.0	15.5	2.6	0.5	9.1	10.4	1.0
MW 12	0.4	1.3	0.8	0.7	19.9	3.2	1.0	4.7	14.1	1.5
MW 13	0.5	2.0	1.5	0.8	21.1	3.1	1.2	2.4	11.6	17.1
TW 1	1.43	0.72	1.60	0.60	7.58	5.73	2.03	9.3	2.8	1.1
TW 2	0.96	0.59	2.20	0.90	22.18	5.59	5.67	7.3	26.4	0.6
TW 3	0.48	1.33	0.65	0.43	5.41	6.45	1.43	6.7	18.5	29.8
TW 4	0.79	0.85	0.98	1.18	4.49	1.74	1.83	4.2	8.0	7.4
TW 5	0.68	0.72	0.98	0.61	2.18	7.87	1.58	2.4	31.3	24.6
TW 6	1.29	0.57	0.52	0.23	13.9	1.73	1.60	4.8	7.2	22.2
TW 7	1.28	1.15	0.35	1.44	4.7	6.17	2.21	0.1	23.9	14.1
TW 8	0.34	0.33	0.46	0.54	6.34	2.17	0.64	30.7	10.5	26.4
TW 9	0.56	0.35	0.54	0.50	5.75	3.25	0.77	6.3	53.7	26.4
TW 10	0.10	0.57	0.73	1.25	—	5.44	1.32	33.1	24.0	8.7
TW 11	0.35	0.70	1.28	1.55	1.67	3.26	5.17	4.7	3.4	26.9
TW 12	0.79	0.40	0.71	0.25	4.26	0.73	5.94	14.3	16.9	5.9
TW 13	0.27	0.93	1.11	0.41	3.56	5.94	0.50	1.3	16.5	15.1
TW 14	0.55	0.50	0.86	0.27	7.12	8.83	2.47	1.8	20.3	70.0
TW 15	0.49	0.82	0.62	0.94	8.46	3.46	1.10	11.2	11.2	25.7
TW 16	1.88	0.33	0.51	0.49	1.56	2.51	0.34	3.7	19.9	18.4
TW 17	1.68	0.50	1.02	0.73	9.86	6.75	4.60	0.9	2.9	0.1
TW 18	0.55	0.95	0.97	0.45	5.75	3.40	0.54	0.7	18.2	9.0
TW 19	0.78	1.48	1.44	0.53	9.65	3.40	4.86	16.6	8.3	28.8
TW 20	0.28	0.41	1.38	0.53	1.64	1.37	0.67	3.1	13.1	15.5
TW 21	1.12	1.14	1.36	1.95	1.63	4.41	0.72	11.9	15.7	35.4
TW 22	1.45	1.48	0.79	1.91	7.13	2.51	2.05	1.1	7.3	2.8
TW 23	1.23	0.65	0.90	0.38	0.34	1.41	1.65	3.4	8.2	25.5
TW 24	3.37	0.88	1.22	1.01	1.93	2.36	2.47	2.5	15.1	7.8

RSD (%) was generated by AAS novAA 400 software, RSD (%) for other minerals were not determined.

**Table 6:** Mineral contents in packaged drinking water (DW), mineral water (MW), and tap water (TW) samples from Malaysia and other countries.

Country		Na	Mg	K	Ca	Fe^a^	Cu^a^	Zn^a^	Cr^a^	Mn^a^	Ni^a^	As^a^	Cd^a^	Pb^a^	F	Cl	NO_3_	SO_4_
*DW*																		
Malaysia (*n* = 14)^b^	Mean	2.22	1.82	1.01	0.36	34.51	2.99	1.19	0.4	5.14	0.55	0.38	0.49	1.28	0.24	19.71	0.56	6.54
	Min	Tr	Tr	Tr	Tr	17.2	0.2	Tr	Tr	ND	0.1	Tr	0.4	Tr	Tr	Tr	0.1	Tr
	Max	10.68	12.29	3.8	1.67	43.3	7.3	2.3	2.56	31.0	2.09	1.68	0.85	3.92	0.71	42.41	2.27	52.02
Turkey study (*n* = 3)^c^	Mean	1.87	2.2	1.2	3.1	0.21	—	0.34	0.23	—	0.15	0.46	0.34	0.23	0.07	2.03	1.93	5.33
	Min	0.8	0.1	0.1	0.0	ND	ND	ND	0.17	ND	0.14	0.12	0.29	0.21	0.04	Tr	0.9	Tr
	Max	3.8	3.5	3.2	6.9	0.64	ND	1.03	0.26	ND	0.17	1.05	0.42	0.24	0.09	5.1	3.1	11.0
Europe (*n* = 40)^d^	Mean	13	16	—	60	—	—	—	—	—	—	—	—	—	—	—	—	—
	Min	1	1	—	4	—	—	—	—	—	—	—	—	—	—	—	—	—
	Max	56	110	—	145	—	—	—	—	—	—	—	—	—	—	—	—	—
North America (*n* = 28)^d^	Mean	4	8	—	18	—	—	—	—	—	—	—	—	—	—	—	—	—
	Min	0	0	—	0	—	—	—	—	—	—	—	—	—	—	—	—	—
	Max	15	95	—	76	—	—	—	—	—	—	—	—	—	—	—	—	—
China (*n* = 3)^e^	Mean	14.69	6.82	1.56	50.84	—	—	0.009	0.001	—	—	—	0.001	—	—	—	—	—
	Min	2.33	2.62	0.81	4.7	—	—	0.005	ND	—	—	—	ND	—	—	—	—	—
	Max	31.51	13.14	2.35	92.8	—	—	0.016	0.003	—	—	—	0.001	—	—	—	—	—
Egypt (*n* = 3)^e^	Mean	88.69	19.54	5.51	59.38	—	—	0.002	0.004	—	—	—	0.001	—	—	—	—	—
	Min	51.41	11.74	5.03	46.18	—	—	0.002	ND	—	—	—	ND	—	—	—	—	—
	Max	162.35	32.94	6.32	66.28	—	—	0.002	0.011	—	—	—	0.002	—	—	—	—	—

*MW*																		
Malaysia (*n* = 13)^b^	Mean	10.17	5.84	2.91	4.46	11.55	12.77	4.79	—	31.53	1.5	3.2	0.36	0.26	0.41	34.46	1.16	9.35
	Min	2.4	0.72	0.6	0.59	Tr	1.5	0.4	Tr	ND	0.28	Tr	0.45	Tr	Tr	27.16	0.12	ND
	Max	23.68	24.03	5.95	25.06	60.5	16.9	24.3	Tr	68	6.88	13.51	0.45	1.25	2	58.58	2.84	18.74
Turkey (*n* = 67)^c^	Mean	9.21	3.23	0.47	25.82	2.29	0.31	10.0	0.64	0.99	0.53	1.77	0.37	0.21	0.11	3.23	3.01	6.46
	Min	0.1	0.1	0.1	0.3	ND	ND	ND	0.14	ND	0.09	0.12	0.29	0.21	Tr	Tr	0.9	0.0
	Max	76.8	19.0	5.3	50.9	48.88	6.78	364.8	6.4	47.96	7.48	30.63	1.36	0.32	0.69	23.3	14.2	62.0
North America (*n* = 9)^d^	Mean	371	24	—	100	—	—	—	—	—	—	—	—	—	—	—	—	—
	Min	36	1	—	3	—	—	—	—	—	—	—	—	—	—	—	—	—
	Max	1095	130	—	310	—	—	—	—	—	—	—	—	—	—	—	—	—
Italy (*n* = 371)^f^	Mean	76.53	26.02	10.42	97.53	182.3	—	45	0.1	627.3	0.0	3.5	0.38	350	0.57	96.95	5.51	113.82
	Min	Tr	Tr	0.09	0.8	0.0	Tr	Tr	Tr	Tr	Tr	Tr	Tr	Tr	Tr	0.15	Tr	0.3
	Max	5051.43	328.4	300	864	4000	Tr	180	2	9800	0.0	7	2	3500	8.4	8055.8	47.49	1918
Various countries (*n* = 66)^g^	Mean	—	—	—	—	—	—	—	—	—	—	—	—	—	—	—	—	—
	Min	ND	ND	ND	ND	—	—	—	—	—	—	—	—	—	ND	ND	ND	ND
	Max	227	170.5	50.5	468.6	—	—	—	—	—	—	—	—	—	2.62	214.1	38.1	1039

*TW*																		
Malaysia (*n* = 24)^b^	Mean	4.3	1.1	3.2	6.65	58.29	8.54	34.71	0.04	25.38	0.91	0.81	0.41	0.28	0.39	20.19	1.39	10.22
	Min	1.22	0.07	0.9	0.54	23.0	1.0	2.0	Tr	Tr	0.26	0.16	0.36	Tr	0.14	5.02	0.33	1.06
	Max	15.03	4.11	6.61	23.1	330.0	26.0	358.0	0.88	91.0	2.63	6.14	0.85	3.8	0.71	48.04	11.0	31.22
Japan (*n* = 3)^e^	Mean	12.7	4.62	1.26	32.0	10.2	—	6.36	—	—	—	—	—	—	—	—	—	—
Kazakhstan (*n* = 3)^e^	Mean	9.59	4.17	1.3	24.8	35.3	—	218	—	—	—	—	—	—	—	—	—	—
Egypt (*n* = 6)^h^	Mean	34.9	14.0	5.84	33.9	73.3	4.59	83.2	7.64	5.2	2.53	—	0.04	0.18	0.25	45.29	0.03	69.06
	Min	33.7	11.7	5.58	30.5	67.2	4.46	11.4	6.99	3.9	2.21	ND	0.03	0.14	0.24	28.36	ND	59.96
	Max	36.0	16.2	6.09	37.2	79.3	4.72	155.0	8.28	6.5	2.85	ND	0.05	0.21	0.26	62.21	0.03	78.16

All data were presented as mean, min, and max (mg/L). ^a^Concentration of minerals in *μ*g/L. Sources: ^b^Present study; ^c^Güler and Alpaslan [24]; ^d^Azoulay et al. [25]; ^e^Chiba et al. [26]; ^f^Naddeo et al. [27]; ^g^Lau and Luk [23]; ^h^Saleh et al. [22].

## References

[1] Chan NW (2004). *Managing Water Resources in the 21st Century: Involving All Stakeholders Towards Sustainable Water Resources Management in Malaysia*.

[2] Pillay M, Hoo T, Chu KK (2001). *Drinking Water Quality Surveillance and Safety in Malaysia for WHO Workshop on Drinking Water Quality, Surveillance and Safety. Country Report*.

[3] Jahi JM (2001). The important of social and economic aspects in integrated drainage basin management system. *Malaysian Journal of Environmental Management*.

[4] Rosborg I, Nihlgård B, Gerhardsson L, Sverdrup H (2006). Concentrations of inorganic elements in 20 municipal waters in Sweden before and after treatment—links to human health. *Environmental Geochemistry and Health*.

[5] Sayre IM (1988). International standards for drinking water. *Journal of the American Water Works Association*.

[6] Pip E (2000). Survey of bottled drinking water available in Manitoba, Canada. *Environmental Health Perspectives*.

[7] Holler AC (1974). Corrosion of water pipes. *Journal of the American Water Works Association*.

[8] Lerner DN (1986). Leaking pipes recharge ground water. *Ground Water*.

[9] Cookson J, Snowberger M, Tomanio J, Staff N (2010). Cost of water: the water issue. *National Geography*.

[10] Ferrier C (2001). Bottled water: understanding a social phenomenon. *AMBIO: Journal of the Human Environment*.

[11] Nardone R (2003). Like oil and water: the WTO and the world's water resources. *Connecticut Journal of International Law*.

[12] Rosborg I, Gerhardsson L, Nihlgård B (2003). Inorganic constituents of well water in one acid and one alkaline area of South Sweden. *Water, Air, and Soil Pollution*.

[13] United States Environmental Protection Agency (USEPA) (1991). *Methods for the Determination of Metals in Environmental Samples. EPA Method 600/4-91-010 supplemented by EPA 600/R-94-111*.

[14] Mahan KI, Leyden DE (1983). Simultaneous determination of sixteen major and minor elements in river sediments by energy-dispersive X-ray fluorescence spectrometry after fusion in lithium tetraborate glass. *Analytica Chimica Acta*.

[15] Association of Official Analytical Chemists (AOAC) (2002). *AOAC Requirements for Single Laboratory Validation of Chemical Methods, DRAFT 2002-11-07*.

[16] World Health Organization (WHO) (2006). *WHO Guidelines for Drinking-Water Quality, Incroporating First and Second Agenda to Third Edition (vol. 1)*.

[17] European Union (EU) Standards (1998). Council Directive 98/83/EC of 3 November 1998 on the quality of water intended for human consumption. *Official Journal of European Community*.

[18] United States Environmental Protection Agency (USEPA) (2009). *Drinking Water Contaminants*.

[19] Legal Research Board (2008). *Food Act 1983 & Food Regulations 1985*.

[20] Armienta MA, Segovia N (2008). Arsenic and fluoride in the groundwater of Mexico. *Environmental Geochemistry and Health*.

[21] Rosborg I, Nihlgård B, Gerhardsson L, Gernersson ML, Ohlin R, Olsson T (2005). Concentrations of inorganic elements in bottled waters on the Swedish market. *Environmental Geochemistry and Health*.

[22] Saleh MA, Ewane E, Jones J, Wilson BL (2001). Chemical Evaluation of Commercial Bottled Drinking Water from Egypt. *Journal of Food Composition and Analysis*.

[23] Lau O-W, Luk S-F (2002). A survey on the composition of mineral water and identification of natural mineral water. *International Journal of Food Science and Technology*.

[24] Güler C, Alpaslan M (2009). Mineral content of 70 bottled water brands sold on the Turkish market: assessment of their compliance with current regulations. *Journal of Food Composition and Analysis*.

[25] Azoulay A, Garzon P, Eisenberg MJ (2001). Comparison of the mineral content of tap water and bottled waters. *Journal of General Internal Medicine*.

[26] Chiba M, Shinohara A, Sekine M, Hiraishi S (2006). Drinking water quality from the aspect of element concentrations. *Journal of Radioanalytical and Nuclear Chemistry*.

[27] Naddeo V, Zarra T, Belgiorno V (2008). A comparative approach to the variation of natural elements in Italian bottled waters according to the national and international standard limits. *Journal of Food Composition and Analysis*.

